# Secondary Functions of Arginine and Sulfur Amino Acids in Poultry Health: Review

**DOI:** 10.3390/ani10112106

**Published:** 2020-11-13

**Authors:** Fernanda Lima de Souza Castro, Woo K. Kim

**Affiliations:** Department of Poultry Science, University of Georgia, 110 Cedar St., Athens, GA 30602, USA; fefelimasc@hotmail.com

**Keywords:** arginine, coccidiosis, functional amino acids, health, poultry, sulfur amino acids

## Abstract

**Simple Summary:**

Historically, studies with amino acids have focused on protein synthesis and accretion, especially with eggs and meat, whereas less importance has been given to their secondary functions on the metabolism. However, certain amino acids, such as arginine, methionine, and cysteine are precursors for other essential molecules in the immune defense, antioxidant system, cell signaling, and gene expression, and can act as regulators in the growth and development of the animals. Because poultry are subjected to stressful conditions throughout their lives, the use of these amino acids and their secondary functions could beneficiate their general health. This review describes the metabolism of arginine, methionine, and cysteine and how they modulate different tissues, especially during challenging conditions. Arginine supplementation has been shown to modulate musculoskeletal health development, reduce fat accretion, and improve the antioxidant system. Moreover, methionine and cysteine could improve the bone development and have a potential in mitigating the negative effects caused by heat stress. Understanding how these amino acids can ameliorate stressful conditions may provide novel insights about their use as nutritional strategies to modulate the health status of chickens.

**Abstract:**

Amino acids such as arginine, methionine, and cysteine are the precursors of essential molecules that regulate growth and health, being classified as functional amino acids. This review describes the metabolism of arginine and the sulfur amino acids and how they modulate, directly or indirectly, different tissues. Emphasis is placed on their effects in supporting health during challenging conditions, such as heat stress and *Eimeria* infection. The use of arginine has been shown to reduce abdominal fat pad in ducks and increase lean tissue and bone mineral density in broilers. Additionally, the sulfur amino acids have been shown to improve bone development and are beneficial during heat stress. The use of L-methionine increased the cortical and trabecular bone mineral densities, in laying hens. Moreover, the dietary inclusion of these amino acids could reduce the damage caused by *Eimeria* spp. infection by regulating the antioxidant system and cell repair. Understanding how these amino acids can mitigate stressful conditions may provide us novel insights of their use as nutritional strategies to modulate the health status of chickens.

## 1. Introduction

Over 700 amino acids (AA) can be found in nature; however, only 20 of them have been recognized as building blocks for proteins in cells [1,2]. Historically, the AA studies have focused on protein synthesis and accretion, whereas less importance has been given to their secondary functions on the metabolism. However, certain AA are precursors for other essential molecules in immune defense, antioxidant system, cell signaling, and gene expression; and can act as regulators in the growth, development, and reproduction of the animals, as reviewed by Wu et al. [2,3]. Because of their importance in the metabolism, Wu [4] proposed the new concept of functional AA, which included arginine (Arg), methionine (Met), and cysteine (Cys).

Arginine, an essential amino acid for birds [1], has been associated with the synthesis of protein and other metabolically-important molecules, such as creatine, nitric oxide, glutamate, polyamines, proline, and glutamine [5,6]. Several studies have shown that Arg has a positive effect on performance traits, such as body weight, carcass yield, and fat reduction in poultry [7,8,9,10,11]. Additionally, as a functional AA, Arg supplementation has also been shown to alleviate oxidative stress and improve antioxidant capacity [12], immunity [13,14], and bone and muscle development [9,10,15]. Therefore, Arg is important for multiple physiological pathways in poultry.

Methionine is also classified as an essential and functional AA [16]. Because plant protein sources are low in Met and birds have a high Met requirement to support the rapid growth, Met is considered the first limiting AA in corn and soybean-based diets [17]. Moreover, Met can be irreversibly converted to Cys [18]. Thus, Cys is classified as a semi essential functional AA [16]. Since the supplementation of Met can also provide Cys, we often consider a specific requirement for Met and another one for Met+Cys (total sulfur amino acids, TSAA) [18]. The main metabolic functions associated to the TSAA are: key AA for protein synthesis, methyl group donors through formation of S-adenosylmethionine, sulfur donors, and precursors of important intermediates in metabolic pathways such as carnitine, polyamine, reduced glutathione, and taurine [17,18].

Although much research in mammals has been performed to evaluate the roles of Arg and TSAA as regulators of key metabolic pathways to improve health, growth, and development, the research addressing the functional properties of these AA in chickens remains scarce. Furthermore, the requirements for Arg and TSAA for broilers and laying hens available in the literature considerably vary and are usually extrapolated from studies performed under comfort conditions. However, different stressors, such as environmental temperatures and intestinal diseases, could impact the availability and metabolic needs of these AA [17,19,20], being beneficial to maintain overall bird’s health from their secondary functions. Therefore, in this review, we discuss the functional properties of Arg and TSAA in poultry. Moreover, the focus of this review is on the potential use of these AA as nutritional strategies to modulate the poultry health in stressful conditions, such as *Eimeria* spp. infection and heat stress.

## 2. Arginine

### 2.1. Arginine Metabolism

**Figure 1:**
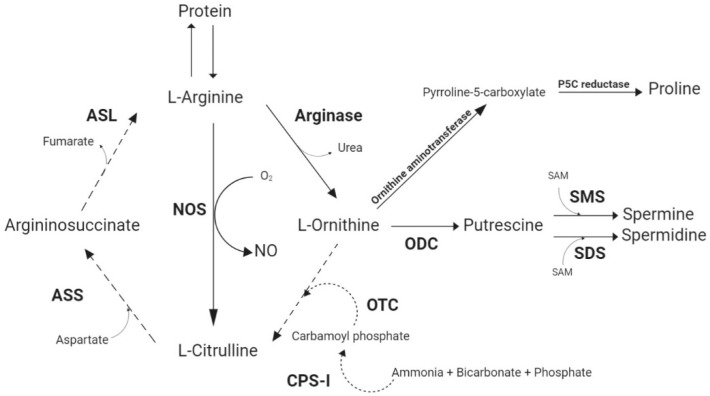
Arginine metabolism. Dotted lines represent pathways that are inactive or less active in birds than in mammals. CPS-I—carbamoyl phosphate synthase I; OTC—ornithine transcarbamylase; ASS—argininosuccinate synthetase; ASL—argininosuccinate lyase; NOS—nitric oxide synthase; ODC—ornithine decarboxylase; SMS—spermine synthase; SDS—spermidine synthase.

Arginine (Arg) is an essential amino acid (AA) required by animals to support protein synthesis, growth, and biological functions [3]. For mammals, which are ureotelic animals, Arg can be obtained through the diet or be synthesized in the urea cycle from metabolically generated ammonia, ornithine, citrulline, and aspartate in the liver and kidney [21,22]. The Arg de novo synthesis is highly dependent on the concentration and activity of the key enzymes such as carbamoyl phosphate synthase I (CPS-I), ornithine transcarbamylase (OTC), argininosuccinate synthetase (ASS), and argininosuccinate lyase (ASL) [6,21] (Figure 1).

However, unlike mammals, the urea cycle in poultry is not entirely functional as they use uric acid as the main nitrogenous waste form (uricotelic animals). Tamir and Ratner [23] investigated the activity of enzymes involved in the urea cycle and Arg metabolism in the liver, kidney, pancreas, intestinal tract, and spleen of chicks. These authors did not detect mitochondrial CPS-I in any of the studied tissues, and a low activity of OTC was detected in the kidney. Therefore, poultry cannot synthesize Arg de novo from ornithine and ammonia. Furthermore, these authors also detected low activities of ASS and ASL in the spleen, kidney, pancreas, and intestinal tract. According to Su and Austic [24], L-citrulline can be converted, to a limited extent, to Arg in the macrophage of birds, serving as a precursor of nitric oxide (NO). However, this sparing effect of citrulline over Arg is not enough to fulfil the Arg need in a growing bird. Therefore, poultry are highly dependent on Arg dietary supply. Since the formation of uric acid does not depend on Arg, the dietary Arg in chicks is used for synthesis of protein and as a precursor of metabolically important molecules, e.g., NO, glutamate, polyamines, and proline through specific pathways initiated by the enzymes arginase and NO synthases (NOS) [5,6].

Arginase is found in microphages, liver, and kidney, where it has its highest activity [25,26]. Ornithine, generated by the arginase pathway, can be decarboxylated by ornithine decarboxylase (ODC) to form putrescine. Putrescine, in turn, can be irreversibly converted into spermine and spermidine [27]. Alternatively, ornithine can be converted to pyrroline-5-carboxylate (P5C) and then converted to proline [28]. The polyamines and proline are associated to cell proliferation, cell repair, and wound healing [21].

NOS catalyzes the conversion of Arg into NO and citrulline, and it is believed that this is the only physiological path for NO production with Arg as the only substrate for all the isoforms of NOS [21]. Because the production of NO depends on the availability of Arg, NOS have to compete for Arg with other Arg-degrading enzymes, such as arginase [29]. However, in endothelial cells, macrophages, and specific cell types, the major proportion of Arg degradation through NOS can vary according to the stimuli [21]. The importance of NO as a modulator for immunity, energy metabolism pathways, gene expression, and blood circulation has been extensively reviewed in the literature [6,30], and it is known to be a versatile and indispensable molecule.

### 2.2. Arginine: Role in Fat, Bone, and Muscle Metabolism

Studies in poultry [7,8,10,15] have suggested that dietary L-Arg supplementation could be effective in reducing body fat deposition. Wu et al. [7] reported a reduction in abdominal fat pad by 4.9% accompanied by lower activity of the enzymes malate dehydrogenase (MDH), glucose-6-phosphate dehydrogenase (G-6-PDH), and fatty acid synthase (FAS) in ducks when 1.0% of Arg was added to the diet. Additionally, Fouad et al. [8] observed a significant reduction in abdominal fat as well as a downregulation of the FAS and an upregulation of the carnitine palmitoyl transferase I (CPT-I) expression in broilers fed 0.25 and 1.0% of Arg compared to the non-supplemented group (1.57 and 1.46% vs. 1.98%). These enzymes participate in the lipogenesis process, indirectly by generating reducing equivalents (MDH and G-6-PDH) and directly by synthesizing fatty acids (FAS), and are involved in the β-oxidation pathway (CPTI) [7,31]. Therefore, the modulation of these enzymes by Arg supplementation could favorably reduce fat accumulation in poultry.

Arginine was shown to act as a secretagogue for insulin [32] and growth hormone (GH) [33]. Moreover, these hormones were shown to act in synergism to stimulate insulin-like growth factor-I (IGF-I) production in chicken hepatocytes [34]. Arg can induce, directly through GH and indirectly through IGF-I, anabolic effects on the metabolism of skeletal muscle. These effects include the stimulation of the aggregation of myofibrillar protein [35] and proliferation and differentiation of satellite cells [36]. In chickens, IGF-I was able to control the growth and feed efficiency partially by modulating the rate of protein breakdown [37], stimulating protein synthesis [38], and reducing protein degradation [37]. Additionally, Arg is also a precursor for the synthesis of creatine, which is involved in muscle protein metabolism [6]. The Arg supplementation increased creatine production in chickens [39], and the supplementation of diets with creatine upregulated the expression of IGF-I gene, consequently increasing leg weight in broilers [40]. Fernandes et al. [9] reported an increase in breast weight and thickness, as well as increased myofiber diameter with increasing dietary Arg levels in broilers during the initial phase (1–21 days). In agreement with the previous study, Corzo et al. [15], Wu et al. [7], Jankowski et al. [11], and Castro et al. [10] reported an increase in carcass yield in broilers, ducks, pectoralis major yield in turkeys, and an increase in total and lean tissue deposition of 21% in broilers supplemented with Arg, respectively. Furthermore, in ovo injection of Arg (100 µg/100 µL/egg) at day 14 of incubation up-regulated myoblast determination protein (MyoD) and myogenin proteins, promoting muscle mass growth of chicks [41].

Additionally, the bone development and health could be improved through the actions of GH, IGF-I, and NO following Arg supplementation. In vitro, Arg supplementation directly stimulated IGF-I at both mRNA and protein levels and increased collagen expression and synthesis in mouse osteoblast-like cells [42]. It has been suggested that a large portion of the IGFs produced by osteoblasts can be incorporated into the bone matrix and stored in the bones for a delayed regulatory action [43]. Both IGF-1 and GH have an anabolic effect on bone tissue, promoting the growth plate-mediated bone growth [44], the differentiation of osteoblast precursors in bone marrow, proliferation of osteoblasts, and increase in type I collagen and matrix formation in bones [45], resulting in increased mineralization. Castro et al. [10] reported an increase in bone mineral density of 16% in broilers supplemented with Arg. Moreover, in vitro, Lys and Arg supplementation increased the production of type I collagen and NO in human osteoblast cultures [46]. Osteoblast-like cells have been shown to express both constitutive and inducible NOS, which could act as a local NO producer in the bone [47]. The local NO production in bones has been shown to favor wound and bone fracture healing in rats [48]. However, the effect of NO on bone development appears to be dependent on its concentration. The slow release and low concentrations of NO have been shown to stimulate the replication of osteoblasts and increase the alkaline phosphatase activity in vitro [49], whereas the rapid release and high levels of NO inhibited the osteoblast proliferation and induced apoptosis [49,50]. These findings suggest that NO has a biphasic effect on bone formation, which might explain the pathophysiology of bone mass loss related to increased inflammatory status [51,52,53].

## 3. Total Sulfur Amino Acid

### 3.1. Total Sulfur Amino Acid Metabolism

**Figure 2:**
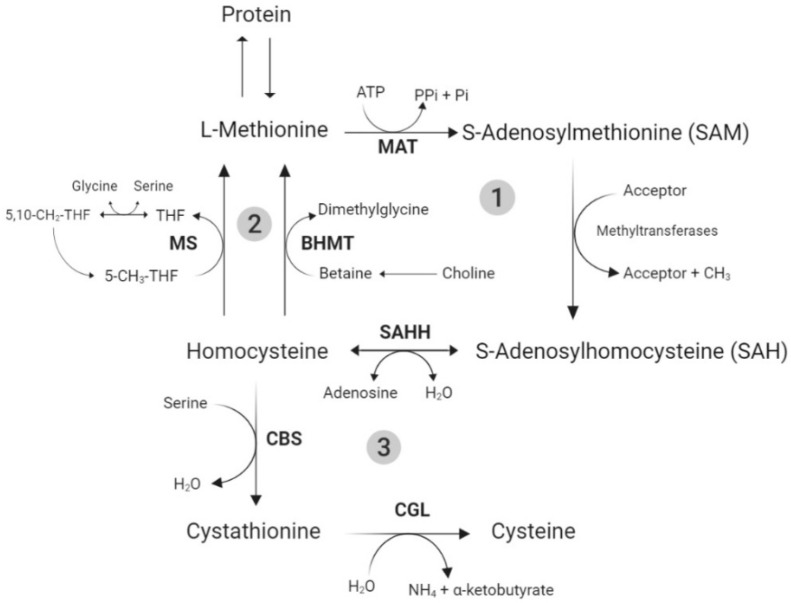
Methionine metabolism. 1—transmethylation, 2—remethylation, 3—transsulfuration. MAT—methionine adenosyltransferase; SAHH—S-adenosylhomocysteine hydrolase; MS—methionine synthase; BHMT—betaine: homocysteine methyltransferase; CBS—cystathionine β-synthase; CGL—cystathionine-γ-lyase (adapted from Brosnan and Brosnan [54]).

The metabolism of Met and Cys, the TSAA, is directly related to the pathway of other molecules, such as homocysteine, taurine, glycine, glutathione (GSH), and polyamines, as previously reviewed in the literature [18,54,55]. In summary, the TSAA metabolic pathway can be divided into three main steps: transmethylation, remethylation, and transsulfuration (Figure 2).

The L-Met, coming from the diet or muscle catabolism, enters the transmethylation step, which begins with the activation of L-Met to S-adenosylmethionine (SAM). After SAM donates its methyl group to an acceptor (e.g., AA residues in proteins, DNA, and RNA), it becomes the coproduct S-adenosylhomocysteine (SAH) [18], which is then hydrolyzed to homocysteine, marking the end of transmethylation. From that point on, homocysteine can go through either the remethylation or the transsulfuration pathways.

The remethylation process ultimately results in the de novo synthesis of L-Met, through the addition of a methyl group to homocysteine. The transsulfuration pathway, which only occurs in the liver, kidney, intestine, and pancreas, consists of the conversion of homocysteine and serine to Cys [18]. It is an irreversible pathway and results in Met catabolism. The Cys can be further oxidized, incorporated to protein, and used in the synthesis of taurine and GSH [56].

Several antioxidant and modulatory functions have been attributed to taurine [57], which could be beneficial during an oxidative stress situation. Moreover, GSH is formed from Cys, glutamate, and glycine; and Cys is considered to be the most limiting AA in this process [58]. The GSH synthesis occurs virtually in all cell types; however, the liver is the major producer and exporter of GSH. The GSH can be, enzymatically (glutathione peroxidases) or nonenzymatically, oxidized by ROS to glutathione disulfide (GSSG). GSSG can, in turn, be reduced back to GSH by glutathione reductase, or be eliminated from the cell. These reactions constitute the glutathione redox cycle [59,60].

Because Met can be irreversibly converted to Cys, the Cys need can be met by supplementing Met, while the requirement for Met can be satisfied only by Met supplementation to the diet [18]. Thus, it is commonly accepted that there is a specific requirement for Met and another one for TSAA.

### 3.2. Total Sulfur Amino Acids: Role in Heat Stress and Bone Metabolism

Several potential stressors have been identified to occur during the birds’ lives, such as temperature stress either by cold [61] or heat [62], and stocking density [63]. High environmental temperatures with high relative humidity (RH) have deleterious effects on the performance of livestock animals, leading to economic losses [64]. In poultry, these deleterious effects are associated to a reduction in welfare, livability, growth performance, egg production, egg weight, and eggshell quality [65]. Several physiological and behavioral changes have been observed when birds are subjected to high environmental temperatures that can lead to changes in nutrient digestibility and absorption. A reduction in protein and Met digestibility was observed in broilers subjected to heat stress (HS) [66,67,68,69]. This decrease was possibly due to (1) a reduction in capillary blood flow to the digestive system and increase to the periphery to facilitate heat exchange [70], interfering with the nutrient influx to intestinal cells and the transport of the absorbed nutrients to the liver, (2) changes in the proteolytic enzymatic activities in the digestive tract [67,71,72], and (3) increase in potential intestinal damage [72]. Thus, HS could considerably limit protein and amino acid utilization in poultry. Changes in Met absorption due to HS were reported in the literature. Dibner et al., [73] and Knight et al. [74] studied, in vitro, the intestinal passage of DL-Met and epithelial uptake with radiolabeled ^14^C-DL-Met in intact small intestine samples from birds subjected to cyclic chronic HS and thermoneutral conditions. Both studies observed a decrease in DL-Met uptake in HS samples, suggesting a shift from the energy sodium-dependent (ESD) transporters to the energy sodium-independent (ESI) transporters. Therefore, the changes in digestion and absorption due to HS could alter the TSAA use by the birds, leading to different requirements.

Moreover, the HS ultimately leads to cellular oxidative stress in poultry, and the TSAA supplementation could mitigate the negative effects of HS by improving the antioxidant capacity. A study conducted by Del Vesco et al. [75] showed that the supplementation with the adequate and excessive doses of DL-Met upregulated the expression of glutathione synthetase and glutathione peroxidase-7 genes in broilers acutely exposed to HS. Additionally, the in ovo supplementation of TSAA in chick embryos subjected to HS from 10 to 18 days of incubation increased the GSH levels and the total antioxidant capacity in the serum, heart, pectoral muscle, small intestine, liver, and kidney at hatch [76].

Another important economic and welfare issue faced by the poultry industry is related to bone health. A survey conducted with end-of-lay hens reported that approximately 29% of laying hens had at least one broken bone during their lifetimes in Europe [77]. Moreover, approximately 85% of laying hens per flock have been affected with keel bone damage [78], whereas 27% of broilers were affected by locomotor disorders in the United Kingdom [79]. Therefore, nutritional interventions have been explored to minimize the development of skeletal disorders [80]. The dietary protein is essential for bone health because it is a component of the organic structural bone matrix and can influence the serum concentration of IGF-I [81]. The IGF-I is a key regulator in bone growth, mainly by promoting the differentiation of osteoblast precursors, stimulating the proliferation of osteoblasts in the bones, and increasing the production of type I collagen [45]. The supplementation of 0.24% of DL-Met in broilers resulted in higher IGF-I and GH receptor expression in the liver [82], whereas the in ovo injection of TSAA increased the expression of IGF-I in the jejunum, heart, pectoral muscle, and liver at hatch [76]. Thus, the TSAA supplementation may be an important regulator of IGF-I synthesis and, consequently, be beneficial in improving growth and bone development in chickens.

Castro et al. [83] have reported that the supplementation of 0.22% of L-Met (0.74% of TSAA), in laying hens, led to an increase in the cortical and trabecular BMD of 6.4 and 4.7%, respectively, compared to a deficient diet. Additionally, Castro et al. [84] demonstrated that pullets fed diets deficient in TSAA had lower bone ash, bone tissue volume, and lower bone mineral content than pullets fed the TSAA levels as recommended by the breeder guideline. Moreover, the TSAA levels could be a limiting factor for the non-collagenous protein (NColP) synthesis in the bones. The NColP are an integral component of the bone’s organic matrix, exhibiting multifunctional roles in the bone quality. It has been shown that NColP influence bone remodeling and mineralization and are structurally important [85]. The most prevalent NColP, accounting for approximately 2% of total protein in a developing bone, is osteonectin [86]. Osteonectin is a calcium-binding glycoprotein, rich in Cys [87], synthesized by osteoblasts and involved in the remodeling and maintenance of bone mass in vertebrates [88]. Therefore, TSAA deficiency could negatively affect the NColP synthesis in the bones.

## 4. Arginine and Total Sulfur Amino Acids Utilization under an *Eimeria* spp. Infection

Over the years, the acknowledgement of the importance of nutrition to the gastrointestinal tract (GIT) health and function has increased, and Arg and TSAA were pointed out for their essential influence on GIT protection and development. In adult rats and adult humans, 38–40% of the luminal absorbed Arg is catabolized in a single pass and utilized by the intestinal mucosa, whereas the remaining 60% enters the circulation intact [89,90]. According to Stoll et al. [91], in piglets, approximately 52% of the dietary intake of Met is sequestered in first-pass utilization and metabolized by the intestines. Additionally, a higher Met requirement was observed in piglets fed enterally than parenterally [92], and there is evidence that Met catabolism in the intestinal cells is not quantitatively significant [1].

These findings suggest a high intestinal metabolism of dietary Arg and Met, and that the GIT might have its own requirement for these AA, which should be considered when considering their supplemental levels. This requirement could be related to energy generation, protein incorporation, or polyamine, Cys, and GSH synthesis [93]. Moreover, the Arg and TSAA absorption also takes place in the GIT. Therefore, any factors that disrupt the normal GIT function and health may interfere with the use of Arg and TSAA by the animal. Several infectious and non-infectious agents can affect the GIT health in poultry, such as mycotoxins, bacterial infections, viruses, and coccidiosis.

Coccidiosis, caused by a protozoa of the genus *Eimeria*, is one of the most important parasitic diseases in poultry production. It is commonly accepted that there are seven species of *Eimeria* which can infect chickens [94]; however, the most prevalent and economically important ones are *E. acervulina*, *E. maxima*, and *E. tenella*. The *Eimeria* reproductive stages take place in the intestinal epithelium, causing severe tissue damage, disruption of digestion and nutrient absorption, inflammation, and increase in oxidative stress [95,96,97,98,99]. The severity of lesions is directly dependent on the number of ingested oocysts by the host and duration of the exposure [94].

The performance loss observed during an *Eimeria* infection in broilers is related to a decrease in feed intake, body weight gain, feed efficiency, and increase in mortality [95]. A meta-analysis has shown that the weight gain of broilers decreased before the reduction in feed intake was observed, indicating that the negative impact of *Eimeria* on performance was not exclusively linked to anorexia [100]. The weight reduction was of 6.1, 10.0, 5.8, and 4.6% in birds infected with *E. acervulina*, *E. maxima*, *E. tenella,* and an *Eimeria* spp. pool, respectively, at a constant feed intake. Therefore, we can assume that, in this kind of challenge, the nutrient utilization can be reduced, and the nutrients absorbed are being redirected to maintain the health status rather than growth.

Since part of the *Eimeria* spp. life cycle takes place in the enterocytes, inflammation, cell death, and sloughing are commonly observed in the intestine of the birds [95]. The intestinal morphology was altered by an *E. avervulina* infection, leading to flattened and thickened villi, with ruptured enterocytes, elongated crypts, and higher turnover rate [101,102]. Moreover, the reduction in the activity of pancreatic and brush border enzymes in areas affected by the *Eimeria* infection has been reported by Russel and Ruff [103], Allen [104], and Adams et al. [105]. Consequently, during the *Eimeria* infection, the digestibility and absorption of nutrients are impaired.

### 4.1. Intestinal Digestion and Absorption

A reduction in L-Met uptake by different intestinal sections, in vitro, was observed in birds challenged with *E. necatrix* [106], *E. acervulina* [107], and *E. maxima* [108] at 7 days post-infection. Persia et al. [109] observed a reduction in the apparent total tract AA digestibility, including Arg, in birds fed a soybean meal diet and acutely infected with *E. acervulina*. The authors also reported that the negative effect of the infection on the AA digestibility was greater during the first 2 days post-infection than in later periods. Rochell et al. [110] studied the apparent ileal digestibility (AID) of AA in chicks inoculated with different doses of *E. acervulina* (2.5 × 10^5^, 5.0 × 10^5^ and 1.0 × 10^6^ sporulated oocysts). The authors observed a linear and quadratic decrease for Arg and Met digestibility, respectively, with increased oocyst dose. The AID reduction was of approximately 2 percentage units for Arg and Met compared to the uninfected birds.

Additionally, Paris and Wong [111] observed that the expression of the transporter systems b^0, +^ and L was down- and up-regulated, respectively, in birds challenged with *E. Maxima*. The b^0, +^ system, which is a Na^+^-independent AA transporter, mediates the inward transport of Cys, Arg, and lysine in exchange with neutral AA [112], whereas the L system, a Na^+^-independent AA transporter, mediates the inward transport of large, neutral, branched or aromatic AA in exchange for the efflux of other AA such as isoleucine, leucine, and Met [113]. The L system shows poor preference for Met uptake but high preference for Met as efflux substrate [113]. Therefore, with lower uptake of Arg by the b^0, +^ system and increase in Met efflux, there is a decrease in intracellular pool of Arg, Met, and other essential AA. The authors hypothesized that this change in transport gene expression may be part of the defense mechanism against the infection, once the decrease in intracellular essential AA would limit the energy and protein production in the cell, leading to inhibition of *Eimeria* spp. replication and potentially cell death. Therefore, it is possible that the Arg and TSAA requirement might be increased due to the intestinal damage and changes in the digestion and absorption processes.

### 4.2. Antioxidant Capacity and Cellular Repair

The reactive oxygen species (ROS) are a group of free radicals generated during the physiological oxygen metabolism [114] and are mainly composed of superoxide (O_2_^−^), hydrogen peroxide (H_2_O_2_) and hydroxyl radical (·OH) [115]. At biological levels, the ROS are used as signaling molecules involved in the homeostasis; however, their production can increase during diseases, such as coccidiosis [96,116,117]. When the production of ROS surpasses the antioxidant defense capacity, it can ultimately lead to lipid peroxidation, proteins and DNA damage, and cell death [118,119]. Recent findings from Liang et al. [120], in rats, strongly suggest that Arg is a free radical scavenger agent, suppressing ROS generation. Furthermore, these authors have shown that oral administration of Arg can increase the activity and expression of key molecules in the antioxidant system: GSH, catalase (CAT), and SOD. These effects seem to be dependent on the Arg availability. The GSH is mainly constituted by L-glutamate, L-Cys, and L-glycine [121]. Because L-glutamate is a product of Arg catabolism through arginase, it is believed that the Arg supplementation may increase the availability of glutamate for GSH synthesis. The Arg supplementation improved the total antioxidant capacity and reduced the malondialdehyde concentration (indicator of lipid peroxidation) in the blood in laying quails and broiler breeders [12,122].

Recent studies have demonstrated the antioxidant properties of Met in poultry and its potential in alleviating oxidative stress. The Met supplementation (5.9 g/kg) in broilers resulted in higher SOD activity in the serum, decreased hepatic malondialdehyde content at 7 days of age, and decreased GSH:GSSG ratio in the liver at day 21 [123]. In 16-week old female turkeys, the supplementation of 0.47% of DL-Met resulted in higher total antioxidant potential in the serum [124]. Moreover, during a mixed *Eimeria* spp. infection model, the supplementation of L-Met was able to increase the GSH levels in the liver of broilers compared to the deficient diet, ameliorating the negative effect of coccidiosis on the antioxidant system [125]. In addition to being involved in the synthesis of GSH, the TSAA are also considered free radical scavenger molecules. Free Met and Cys, as well as surface exposed Met and Cys residues in proteins can be readily oxidized by ROS [126,127]. The oxidized Cys can be reduced through several enzyme systems, while the reduction of the oxidized Met (methionine sulfoxide, MetO) is catalyzed by methionine sulfoxide reductases [126,127]. The oxidation of these AA residues in proteins may be responsible for protecting protein structure and function under oxidative stress. In in vitro studies, Levine et al. [128] observed that about half of the Met residues in the enzyme glutamine synthetase from *E. coli* could be oxidized with little effect on its catalytic effect. Kim et al. [129] also demonstrated, in vitro, that Cys, Met, and taurine have superoxide radical and hydrogen peroxide scavenging effects. Additionally, Cys showed radical scavenging activity similar to ascorbic acid when other free radical molecules were used. Thus, the TSAA antioxidant properties could be further explored in different stressful conditions when supplementing those AA.

Putrescine, spermine, and spermidine are the most common polyamines, which are small aliphatic polycation molecules widely distributed in nature. The formation of these polyamines in birds is dependent on both Arg and Met [27,130]. It has been recognized that polyamines are important in modulating gene expression, cell growth and proliferation, protein translation, cell apoptosis, and can act as antioxidant molecules [130]. Therefore, these molecules could be important during an intestinal challenge and the recovery post-challenge. Furthermore, it is believed that the dietary manipulation of AA can alter the metabolism of polyamines in birds [27], and the supplementation with Arg and TSAA could be beneficial for modulating the polyamine synthesis in the intestine of infected birds.

Rochell et al. [131] found a linear reduction in plasmatic Arg concentration with increasing *Eimeria acervulina* challenge dosage, and, when the highest dose was used (1.0 × 10^6^ sporulated oocysts), this reduction was off 22% compared to noninfected birds. Additionally, the authors have also observed that this plasmatic Arg reduction was accompanied by a 95% increase in plasmatic ornithine. Two theories were provided by the authors to explain these findings: (1) the reduction in plasmatic Arg of infected birds may have been related to increased demand of Arg for NO production, which is a key mediator of the immune response to coccidiosis [132,133]; and (2) the increase in plasmatic ornithine, produced by arginase, can be a precursor for polyamine synthesis [134]. In this context, L-Met is also required in the synthesis of polyamines via SAM, and both AA could improve the intestinal health.

## 5. Conclusions

The supplementation of arginine and the total sulfur amino acids has been shown to improve the musculoskeletal development, the intestinal health, and the antioxidant system of poultry. The mechanism by which these amino acids modulate general health is mainly associated to their function as precursors of key metabolic molecules, such as nitric oxide, creatine, and glutathione, and as secretagogues for growth hormone and IGF-I. Furthermore, the digestion and absorption of both arginine and the total sulfur amino acids were shown to decrease in an *Eimeria* spp. infection, and the supplementation of these amino acids could help to improve the intestinal health during and after the infection. Thus, future studies should address the supplementation of arginine and total sulfur amino acids as nutritional strategies in improving health, especially in birds subjected to stressful conditions including HS and disease infection.
